# Ventilation Prediction for an Industrial Cement Raw Ball Mill by BNN—A “Conscious Lab” Approach

**DOI:** 10.3390/ma14123220

**Published:** 2021-06-10

**Authors:** Rasoul Fatahi, Rasoul Khosravi, Hossein Siavoshi, Samaneh Yazdani, Esmaiel Hadavandi, Saeed Chehreh Chelgani

**Affiliations:** 1 School of Mining Engineering, College of Engineering, University of Tehran, Tehran 16846-13114, Iran; rasoul.fatahi95@gmail.com; 2 Department of Mining, Faculty of Engineering, Lorestan University, Khorramabad 68151-44316, Iran; rlkhosravi@gmail.com; 3 Department of Mining and Geological Engineering, University of Arizona, Tucson, AZ 85721, USA; hosseinsiavoshi@email.arizona.edu; 4 Department of Electrical and Computer Engineering, North Tehran Branch, Islamic Azad University, Tehran 16511-53311, Iran; s_yazdani@iau-tnb.ac.ir; 5 Department of Industrial Engineering, Birjand University of Technology, Birjand 97198-66981, Iran; es.hadavandi@gmail.com; 6 Minerals and Metallurgical Engineering, Department of Civil, Environmental and Natural Resources Engineering, Luleå University of Technology, SE-971 87 Luleå, Sweden

**Keywords:** cement, ball mill, conscious laboratory, random forest, support vector regression

## Abstract

In cement mills, ventilation is a critical key for maintaining temperature and material transportation. However, relationships between operational variables and ventilation factors for an industrial cement ball mill were not addressed until today. This investigation is going to fill this gap based on a newly developed concept named “conscious laboratory (CL)”. For constructing the CL, a boosted neural network (BNN), as a recently developed comprehensive artificial intelligence model, was applied through over 35 different variables, with more than 2000 records monitored for an industrial cement ball mill. BNN could assess multivariable nonlinear relationships among this vast dataset, and indicated mill outlet pressure and the ampere of the separator fan had the highest rank for the ventilation prediction. BNN could accurately model ventilation factors based on the operational variables with a root mean square error (RMSE) of 0.6. BNN showed a lower error than other traditional machine learning models (RMSE: random forest 0.71, support vector regression: 0.76). Since improving the milling efficiency has an essential role in machine development and energy utilization, these results can open a new window to the optimal designing of comminution units for the material technologies.

## 1. Introduction

In the cement industry, grinding is one of the most consuming energy stages in the production units. Around 65% of the total used electrical energy in a cement plant has to be utilized to grind raw materials, coal, and clinker [1,2]. Through the process, many variables can affect the efficiency and productivity of this dry grinding procedure, such as the operating conditions of the separators, airflow through the mill, the aperture size of the mill partitions, feed rate, the hardness of the feed material, and ball sizes in the mill compartments. Understanding the relationships between these variables from the grinding units can play an essential role in efficiently operating cement production lines [3,4,5].

One of the most critical variables in the cement ball mills is ventilation. A mixture of hot and recycled air would deliver and dry the powder in a cement ball mill as a pulverizing system. Thus, ventilation in this system can have a significant effect on the production process. Extensive ventilation can substantially increase the coarse powder, while in the small ventilation, the delivered powder decreases (as a result, the output decreases). Only proper ventilation ensures the max output with qualified powder, and reduces loss [6,7,8]. The multiphase character of flow made the ventilation controlling an essential factor (where recirculation gases, pulverized coal, sand, and other materials are included) [6,7,8,9]. In detail, there are many parameters in a cement ball mill that may influence the ventilation. The ball mill load, the outlet temperature, the hot air pressure, the recycle air pressure, the negative inlet pressure, the different inlet-outlet pressure, and the outlet pressure of the fine and coarse separators are just some of those influential variables [6,7,8,9,10]. However, few studies have explored the possible relationships between operating variables and ventilation for an industrial ball mill in a cement plant [11,12].

For assessing complex relationships among a wide range of variables monitored from the industrial plants, constructing advanced artificial intelligence (AI) models based on the existing data could be a key to better measure the importance of variables. The development of such reliable models as a new concept recently has been called “conscious-laboratory (CL)”. Generating a CL by using a robust AI model can reduce cost, save time, improve the controlling system, and remove scale-up challenges [13,14]. However, few investigations have used AI models in the cement industry to explore correlations among operational variables [15,16], and absolutely no study has considered a CL for analyzing the effect of various operating variables on mill ventilation. This study will introduce a CL developed by Boosted Neural Network (BNN) as a recently construct AI model to fill the gap. For the first time, this work addresses the importance and effectiveness of all monitored variables on the mill ventilation, based on the actual monitored data from line one of the Ilam cement plant (Ilam, Iran) by a BNN model. The BNN prediction results were compared to two other traditional advanced AI models (random forest and support vector regression) for accuracy assessment purposes.

## 2. Materials and Methods

### 2.1. Database

**Figure 1:**
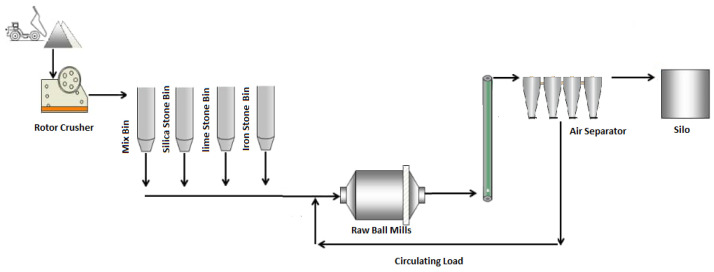
Schematic of ball mill circuit in the Ilam cement plant.

To investigate relationships between various measured variables and ventilation rate, the data were collected from one of the Raw Material ball mill circuits (line 1) of the Ilam cement plant (Figure 1). This plant has 2 lines for cement production (5300 t/d). The ball mill has one component, 5.20 m diameter, and 11.20 m length with 240 t/h capacity (made by PSP Company from Přerov, Czechia). The mill’s rotation speeds are mainly constant (14 rpm), and there is approximately a fixed one-year period of changing liners. Various parameters are monitored in this unit (Table 1). Variables were hourly monitored and were taken into account (when the circuit was stable and balanced). In general, over 2000 records were used for the modeling. Regularly measuring pressure before and after the mill fan (PBMF and PAMF, respectively) can be considered as ventilation factors in a mill.

### 2.2. AI Models

#### 2.2.1. Boosted Neural Network

As a powerful artificial intelligence (AI) model, Boosted Neural Network (BNN) was constructed using neural network experts and ensemble algorithm. BNN promotes the probability of sampling data for training experts in predictive functions. It improves the verification of balance through the training dataset by conducting a wide distribution of inputs and decreasing prediction errors by considering previous experts’ prediction information [17,18,19,20]. It can linearly and nonlinearly examine relationships among a dataset by assessing multivariable sensitivity analyses (MSA), evaluate the sensivity of output to the given inputs, indicate magnitudes, and rank variables based on their importance [20]. In this work, the BNN’s marginal model (MM) was used for the MSA assessments. In the BNN-MM, inputs are ordered based on the size of their overall total effect importance indices [21]. BNN can reduce the objective function (Equation (1)) for the traning step [22], as follows:

(1) $$E_{t}=\sum_{i=1}^{N}{\left(y_{i}-{\hat{y}}_{i}^{t}\right)}^{2}+{\alpha W}_{t}^{T}W_{t}$$

where N is the number of samples in the training dataset, y is the target value, $\hat{y}$ is the predicted value by t-th, experts, and α is the parameter, which is between 0 and 1. $W_{t}$ is the weight vector of t-th neural network expert in the BNN. 

#### 2.2.2. Random Forest

As a tree-based statistical model, random forest (RF) was constructed and developed by Breiman et al. (1993) [23]. A powerful AI machine learning (ML) model can provide low-bias and low-variation outcomes, with highly accurate predictions [24,25,26,27]. In this system, an estimated value generates according to the average of overall trees through the bagging system; different bootstrap data *L(θ)* with size n would be selected from the training set (*L*) with size N. Each tree “*T_L(θ)_*” would be related to the random vector *θ*, which is given for the bagged samples from the main training set *L*. The final predictor “*f*” is the average over the forest (with *y*’*_η_* the estimated response for sample *x_η_* where K is the size of the ensemble) [28,29,30], as follows:

(2) $$y{'}_{\eta }=f\left(X_{\eta }\right)=\frac{1}{K}\sum_{K=1}^{K}\left(T_{L(\theta_{k})}{\left(X_{\eta }\right)}_{1}^{K}\right)$$

#### 2.2.3. Support Vector Regression

Support vector regression (SVR) has been developed based on the structural risk minimization (SRM) principle from statistical learning theory. Regarding the SRM principle, SVR can reduce the risk of overfitting in prediction and generate a compressive model by taking the information of all outputs for the prediction [31,32,33]. SVR can transfer a complex nonlinear regression problem to a linear regression problem in a high dimensional variable space. In other words, a linear function f (SVR function) can be used to formulate the nonlinear relationship between *X_i_* and *Y_i_*, as follows:

(3) f(x)=wφ(x)+b

where *f(x)* shows the predicted value, and the two parameters $w\in {ℜ}^{n_{h}}$ and b∈ℜ must be adjusted. For SRM, empirical risk Equation (4) can be considered where **ε** (**ε**-insensitive) is a precision parameter representing the radius of the tube located around the regression function [33,34].

(4) $$\min R_{e}\left(w,\xi {\;}^{*},\;\xi \right)=\frac{1}{2}{\left|w\right|}^{2}+C\sum_{i=1}^{n}\left(\xi {\;}^{*}+\xi \right)$$

With the following constraints:

(5) $$\{\begin{array}{c} y_{i}-w\varphi \left(x_{i}\right)-b\leq \varepsilon +\xi_{i}\;i=1,\;2,\;3,\;\ldots n\\ -y_{i}+w\varphi \left(x_{i}\right)+b\leq \varepsilon +\xi_{i}\;i=1,\;2,\;3,\;\ldots n \end{array}$$

$$\{\begin{array}{c} \xi_{i}{}^{*}\geq 0\;i=1,\;2,\;3,\;\ldots n\\ \xi_{i}\geq 0\;i=1,\;2,\;3,\;\ldots n \end{array}$$

## 3. Results and Discussion

### 3.1. Variable Importance Measurement

**Figure 2:**
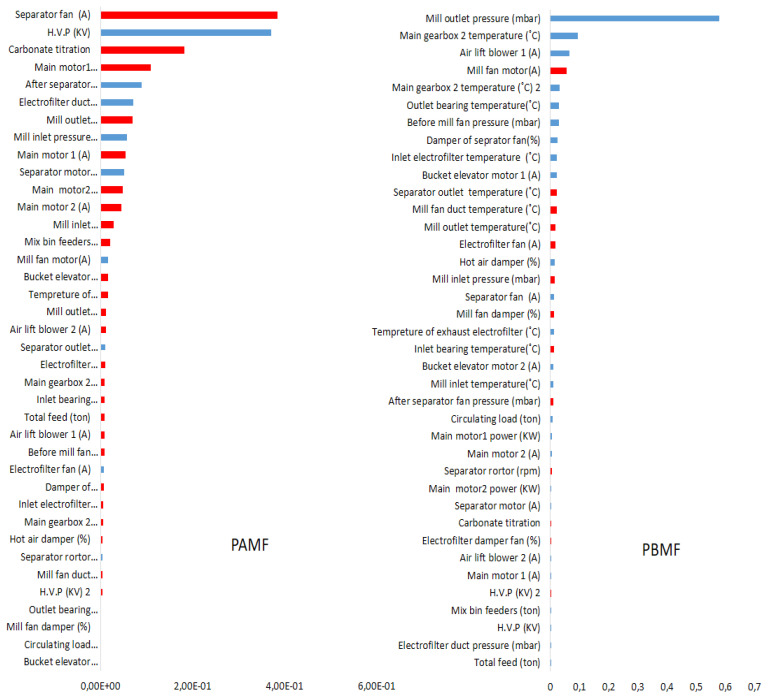
Variable importance measurement for ranking variables. Pearson correlation: Positive (blue), negative (red).

For the variable importance measurement, BNN-MM considers the mean response of the target for each predictor record. The mean is taken over all inputs for the calculation of the importance indices. BNN-MM assessments among monitored variables in the plant indicated that the mill outlet pressure had the highest effectiveness (rank) on the PBMF prediction. There was a positive correlation between these two factors (Figure 2). For the PAMF prediction, the ampere of the separator fan had the highest rank, and by increasing its ampere, the pressure after the mill fan was decreased (a negative relationship) (Figure 2). These relationships could be because fan capacity can limit the separator fan capacity, and pulling more air can increase the separator capacity when the mill separator was not running at its maximum speed [35]. Zachariades (2015) indicated that the differential pressure in the mill is proportional to the primary air fan, which was produced the differential pressure [36].

### 3.2. Prediction

**Figure 3:**
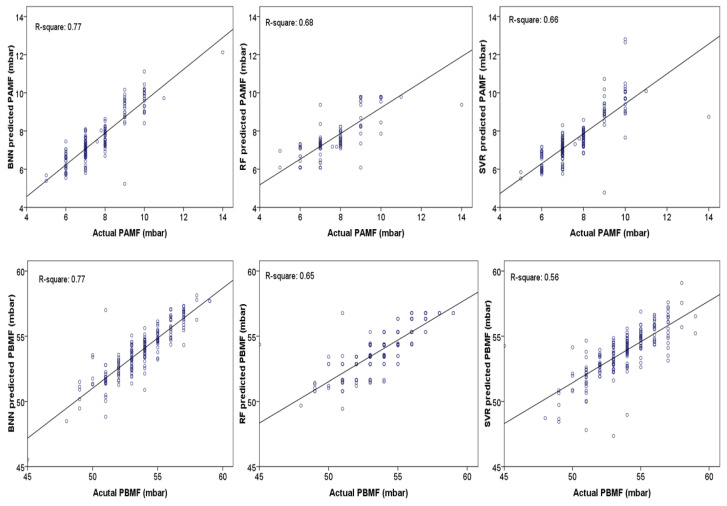
Coefficient of determination (R-square) for the different model outcomes in the testing stage.

The samples of records (1870) were randomly applied for the training stage from the database, and the rest of the records (198) were used for the testing step. For constructing the most accurate predictive BNN model, a trial and error practice was considered to obtain the number of experts. The robust BNN model was generated by the five neural network experts. The developed BNN model expert was a one-layer perceptron neural network with four hidden neurons and a ‘tanh’ activation function. The back-propagation learning algorithm was applied for the training of the experts. The BNN outcomes (Table 2) showed that the developed CL by BNN could comprehensively predict both PAMF and PBMF based on the plant’s monitored parameters. For evaluation purposes, precisely the same databases that were used for constructing the CL-BNN were applied for the development of RF and SVR modeling as typical ML methods. The results (Figure 3) demonstrated that BNN could provide higher accuracy for the PAMF and PBMF prediction than these traditional ML methods. These outcomes highlighted the potential of CL for controlling, sustaining, and estimating other essential variables within cement plants.

## 4. Conclusions

As a virtual laboratory, the development of a conscious-laboratory can be an essential step for maintaining and controlling an industrial plant. For the first time, this study explored the relationships between operational variables and ventilation factors for an industrial cement ball mill, and developed a conscious lab by BNN as a newly generated AI model. Variable importance assessments indicated that the mill outlet pressure and the separator fan’s ampere had the highest importance for predicting ventilation factors. Assessing relationship magnitudes showed that operating variables might have reverse effects on ventilation parameters; however, the mill outlet’s pressure positively correlates with the ventilation factors. In a dry grinding system, the BNN results showed that ventilation, as an efficient factor, could be accurately predicted through a conscious lab procedure. BNN could accurately predict the measuring pressure before and after the mill fan, with R^2^ = 0.77. A comparison between BNN outcomes and random forest/support vector regression, R^2^ < 0.70, approved this newly developed model’s accuracy. These considerable outcomes indeed stressed the possibility of automatic maintenance through cement plants’ operation, where the conscious lab principle showed its reliability for the monitored data assessments.

## Figures and Tables

**Table 1:** Descriptive statistics of monitored variables in the plant.

Variables	Minimum	Maximum	Mean	Std. Deviation
Temperature of exhaust electro filter °C	50	260	119.90	12.85
HVP (1) (KV)	7	596	62.90	129.80
HVP (2) (KV)	31	74	67.00	3.60
Electro filter duct pressure (mbar)	1	41	14.33	1.99
Inlet electro filter temperature °C	44	173	106.14	7.55
Electro filter damper fan (%)	15	100	98.93	7.49
Electro filter fan (A)	22	89	38.75	3.47
Mill fan duct temperature °C	2	115	79.39	7.50
After mill fan pressure (mbar)	4	18	7.52	1.32
Before mill fan (mbar)	39	64	53.88	2.30
Mill fan motor (A)	6	84	62.33	2.48
Mill fan damper (%)	80	100	99.90	1.25
Hot air damper (%)	46	100	57.30	11.82
Separator rotor (rpm)	17	50	22.64	3.92
After separator fan pressure (mbar)	0	109	2.28	2.52
Total feed (ton)	18	230	191.01	17.67
Mix bin feeders (ton)	18	220	180.1	18.95
Damper of separator fan (%)	35	95	51.75	6.79
Before mill fan pressure (mbar)	9	73	14.17	2.48
Separator outlet temperature °C	43	112	73.63	7.79
Separator fan (A)	26	180	29.94	3.45
Separator motor (A)	12	1173	119.56	23.49
Airlift blower2 (A)	15	264	170.84	12.05
Airlift blower1 (A)	17	198	172.76	11.18
Buck elevator motor2 (A)	40	87	54.54	3.15
Buck elevator motor1 (A)	42	72	53.88	4.00
Main motor2 (A)	23	841	241.36	20.50
Main motor1 (A)	205	2369	241.39	47.23
Mill outlet pressure (mbar)	24	369	35.63	7.85
Mill inlet pressure (mbar)	2	14	9.59	0.96
Main gearbox2 temperature °C	12	60	35.79	6.63
Main gearbox1 temperature °C	14	56	40.90	5.97
Outlet bearing temperature °C	30	64	51.37	5.52
Inlet bearing temperature °C	28	59	44.80	4.85
Mill outlet temperature °C	41	124	80.29	7.30
Mill inlet temperature °C	69	498	281.90	22.48
Circulating load (ton)	4	468	143.03	64.47

**Table 2:** Statistical indexes for the outcomes of the training step from different models.

	PAMF		PBMF	
Model	MAE	RMSE	MAE	RMSE
BNN	0.43	0.60	0.77	1.06
SVR	0.45	0.76	0.98	1.46
RF	0.48	0.71	0.91	1.31

## Data Availability

Data available on request due to restrictions e.g., privacy or ethical.
